# Supplementary material for: Single and multi-trait genomic prediction for agronomic traits in Euterpe edulis
Source: PLoS One. 2023 Apr 7;18(4):e0275407. doi: 10.1371/journal.pone.0275407 (PMC10081805; doi:10.1371/journal.pone.0275407)
Supplement: S1 Table — (DOCX) [file pone.0275407.s001.docx]

**Supplementary Table 1-**Genetic (upper diagonal) and phenotypic (lower diagonal) correlation and respective standard errors between the traits RL (Rachis Length), EDF (Equatorial Fruit Diameter), MFB (Fruit Mass per Bunch), NB (Bunch Number) and PY (Pulp yield).

|  | NB | RL | MFB | EDF | PY |
| --- | --- | --- | --- | --- | --- |
| NB | 1 | -0.02 (± 0.33) | -0.33 (± 0.37) | -0.16 (± 0.27) | 0.07 (±0.26) |
| RL | 0.31.(± 0.07) | 1 | 0.29 (± 0.25) | 0.40 (±0.23) | -0.12 (±0.21) |
| MFB | 0.17 (± 0.08) | 0.41 (± 0.07) | 1 | 0.36 (± 0.23) | -0.22 (±0.21) |
| EDF | -0.15 (± 0.08) | 0.14 (± 0.08) | 0.17 (± 0.08) | 1 | -0.21 (± 0.18) |
| PY | 0.03 (± 0.08) | - 0.17 (± 0.12) | -0.14 (± 0.08) | -0.02 (± 0.01) | 1 |
